# Novel Approaches for the Treatment of Patients with Richter’s Syndrome

**DOI:** 10.1007/s11864-022-00973-1

**Published:** 2022-03-16

**Authors:** Andrea Iannello, Silvia Deaglio, Tiziana Vaisitti

**Affiliations:** Functional Genomics Unit, Department of Medical Sciences, University of Torino, Via Nizza 52, 10126 Turin, Italy

**Keywords:** Richter’s syndrome, Targeted therapy, Antibody-drug conjugates, Kinase inhibitors, Monoclonal antibodies

## Abstract

In the last 10–15 years, the way to treat cancers has dramatically changed towards precision medicine approaches. These treatment options are mainly based on selective targeting against signaling pathways critical for or detrimentally activated in cancer cells in cancer cells, as well as exploiting molecules that are specifically expressed on neoplastic cells, also known as tumor-associated antigens. These considerations hold true also in the hematological field where a plethora of novel targeted agents have reached patients’ bedside, significantly improving clinical responses. Chronic lymphocytic leukemia (CLL) is an example of how targeted therapies, such as BTK, PI3K, or Bcl-2 inhibitors as well as anti-CD20 antibodies, have improved patients’ management, even when adopted as frontline treatment. However, these advancements do not apply to Richter’s syndrome (RS), the transformation of CLL into a very aggressive and fatal lymphoma, occurring in 2–10% of patients. RS is usually a fast-growing lymphoma of the diffuse large B cell or the Hodgkin’s variant, with a dismal prognosis. Despite advancements in depicting and understanding the genetic background of RS and its pathogenesis, no significant clinical results have been registered. In the last couple of years, several studies have started to investigate the impact of novel drugs or drug combinations and some of them have opened for clinical trials, currently in phase I or II, whose results will be soon available. This review will present an overview of current and most recent therapeutic options in RS, discussing also how results coming from xenograft models may help in designing and identifying novel treatment opportunities to overcome the lack of effective therapies.

## Introduction

The landscape of lymphomas is currently rapidly changing and becoming more complex, both because of novel subtype classifications based on the genetic and transcriptomic profiles of neoplastic cells [1–5], and due to more treatment options, that are progressively available. These two paths are paving the way for the transition towards personalized approaches where patients will be cured based on cancer cell characteristics to achieve the best response while minimizing side effects.

RS is defined by the WHO classification of tumors of hematopoietic and lymphoid tissues as the development of a high-grade aggressive lymphoma in a previous or concomitant background of CLL [1]. RS is typically a diffuse large B-cell lymphoma (DLBCL) [6, 7], with only a minority of cases (0.5–5%) presenting a Hodgkin’s variant [8, 9]. Even though the prognosis of RS is generally poor, a significant difference is registered when considering the clonal relatedness to the CLL phase. Indeed, clonally related cases show a median survival of approximately 12 months, while clonally unrelated RS are characterized by a median survival of 65 months [10]. Beside the aggressiveness of the disease, an element of poor survival for RS patients is the lack of effective therapies. Indeed, while treatment options for CLL patients have significantly increased with the introduction of targeted agents, such as rituximab, ibrutinib, or venetoclax [11•], resulting in improved OS and different treatment regimens to be adopted depending on the genetic and molecular features of CLL cells, almost nothing has changed for RS patients. In the last couple of years, this gap has started to close with novel agents or drug combination strategies being tested in clinical trials, also thanks to the availability of representative preclinical models.

The incidence rate of RS has been estimated 0.5–1% per year, with an overall incidence in CLL patients of 5–16% [12, 13]. However, a still open point regarding RS incidence is whether treatment regimens adopted in the CLL phase may somehow exert a selective pressure, finally pushing toward RS transformation. Epidemiological results are still controversial, depending also on patient cohorts analyzed. Recent analyses considering treatment-naïve CLL patients who underwent only novel agent therapies showed no increase in the number of RS transformation. On the contrary, when considering relapsed/refractory CLL patients, the incidence raised up to 2–15% following ibrutinib, venetoclax, or idelalisib treatments [14–18].

Results coming from large and prospective cohorts will clarify in the next future the impact of these novel therapies in potential selection of more aggressive clones that can eventually transform into RS.

## Treatment options

Currently, RS patients are treated with the same therapeutic regimens commonly adopted for aggressive B-cell non-Hodgkin lymphomas or de novo DLBCL, mainly based on chemo-immunotherapy and stem cell transplantation (SCT), depending on the fitness of the patients. However, in the former case, only limited efficacy with frequent relapses are registered [19], while SCT can be adopted only in fit RS patients [20, 21]. These limitations require additional investigation of alternative and more effective therapeutic strategies. In the last years, different trials have started with the aim of testing the clinical efficacy of novel compounds or drug combinations. Moreover, generation of RS patient–derived xenograft models has been of help for the designing and preclinical validation of selective therapeutic approaches.

Here, we present a brief overview of the currently available (mainly chemo-immunotherapy and SCT) treatment options for RS patients, moving then to novel agents that are presently under investigation.

### Current treatments

#### Chemo-immunotherapy

Different chemotherapy or chemo-immunotherapy regimens are adopted to treat RS patients. Chemotherapy alone, mainly based on cyclophosphamide, doxorubicin, vincristine, and prednisone (CHOP) administration, results in limited overall response rate (ORR; approximately 20–30%) and a median overall survival (OS) of few months (4–8 months) [22, 23]. Its efficacy is slightly improved when combined with rituximab (R), a human/murine chimeric anti-CD20 monoclonal antibody (mAb). R-CHOP resulted in an ORR of 67% and a median OS of 21 months, three-time longer than CHOP alone [22–24]. On the contrary, no significant clinical improvements compared to chemotherapy alone have been obtained by the CHOP-O treatment, where rituximab was replaced by ofatumumab, another humanized mAb anti-CD20 [25, 26], R-EPOCH (rituximab, etoposide, cyclophosphamide, doxorubicin, vincristine, and prednisone) [27], or R-hyper-CVXD/R-MA regimens, an intensive treatment scheme based on fractioned cyclophosphamide, vincristine, liposomal daunorubicin, and dexamethasone in combination with rituximab alternated to methotrexate and cytarabine plus rituximab [28, 29].

Despite the unsatisfactory results obtained and apart from the ongoing clinical trials (Table 1), the use of chemo-immunotherapy for RS patients remains the frontline therapy, underlining the urgent need for novel and more effective therapeutic strategies.

**Table 1:** Clinical trials enrolling RS patients and evaluating single or combined therapeutic agents

Trial ID	Status	Disease	*N*° of patients	Treatments	Phase	References
NCT03054896	Recruiting	RS	67	Venetoclax, DA-R-EPOCH, R-CHOP	Phase II	[102•]
NCT03899337	Not yet recruiting	CLL, RS	105	Acalabrutinib, R-CHOP	Phase II	[100]
NCT04939363	Recruiting	RS	15	Obinutuzumab, ibrutinib, venetoclax	Phase II	
NCT01171378	Completed	CLL, RS	43	Ofatumumab	Phase II	[25]
NCT03931642	Recruiting	RS	35	R-CHOP, blinatumomab	Phase II	
NCT02576990	Completed	PMBCL, RS	80	Pembrolizumab	Phase II	[41]
NCT04679012	Recruiting	CLL, RS	20	Polatuzumab vedotin, DA-R-EPOCH	Phase II	
NCT04992377	Not yet recruiting	RS	30	R-EPOCH, ibrutinib	Phase II	
NCT04271956	Recruiting	RS	48	Tislelizumab, zanubrutinib	Phase II	
NCT03121534	Active, not yet recruiting	RS	10	Blinatumomab, dexametasone	Phase II	
NCT02846623	Recruiting	CLL, RS	65	Atezolizumab, obinutuzumab, venetoclax	Phase II	
NCT04082897	Recruiting	RS	28	Obinutuzumab, atezolizumab, venetoclax	Phase II	
NCT00309881	Completed	CLL, RS	75	R-CHOP	Phase II	[19]
NCT02420912	Active, not yet recruiting	R/R CLL, RS	72	Ibrutinib, nivolumab	Phase II	
NCT04305444	Recruiting	R/R CLL, DLBCL, FL, RS	120	DTRM-555	Phase II	
NCT02530515	Completed	CLL, RS, PLL	8	Ex vivo–activated autologous lymph node lymphocytes	Phase II	
NCT02332980	Active, not yet recruiting	NHL, CLL, MZL, FL, CLL, RS	65	Ibrutinib, idealisib, [embrolizumab	Phase II	[40]
NCT00452374	Completed	R/R CLL, RS	48	OFAR	Phase I/II	[105]
NCT03534323	Recruiting	CLL, RS	67	Duvelisib, venetoclax	Phase I/II	[106]
NCT04623541	Recruiting	R/R CLL, RS	102	Epcoritamab	Phase I/II	
NCT00304005	Completed	R/R CLL, RS	35	Laromustine	Phase I/II	
NCT00472849	Completed	R/R CLL, RS	92	OFAR	Phase I/II	[107]
NCT02029443	Active, not yet recruiting	CLL, RS	306	Acalabrutinib	Phase I/II	[80]
NCT01217749	Completed	CLL, RS	71	Ibrutinib, ofatumumab	Phase I/II	[98]
NCT05025800	Recruiting	NHL, FL, MZL, MCL, RS	52	ALX148, lenalidomide, rituximab	Phase I/II	
NCT04491370	Recruiting	BL, DLBCL, FL, MCL, MZL, RS	20	ASCT, polatuzumab vedotin	Phase I/II	
NCT02362035	Active, not yet recruiting	FL, CLL, RS, MCL, NHL, MM, BL, MZL	161	Acalabrutinib, pembrolizumab	Phase I/II	
NCT03010358	Completed	FL, MCL, MZL, R/R CLL, RS	24	Entospletinib, obinutuzumab	Phase I/II	
NCT03162536	Recruiting	CLL, MCL, DLBCL, RS, FL, MZL	190	Nemtabrutinib	Phase I/II	
NCT03892044	Recruiting	CLL, DLBCL, RS	44	Duvelisib, nivolumab	Phase I	
NCT04978779	Recruiting	R/R CLL, RS	54	VIP152	Phase I	
NCT04781855	Not yet recruiting	R/R CLL, RS	50	Ibrutinib, ipilimumab, nivolumab	Phase I	
NCT03884998	Recruiting	RS, NHL, FL, LPL, MZL	15	Copanlisib, nivolumab	Phase I	
NCT03778073	Recruiting	NHL, RS	72	Cosibelimab, ublituximab, bendamustina	Phase I	
NCT03113695	Active, not yet recruiting	RS	10	Obinutuzumab, Ienalidomide, HDMP	Phase I	
NCT02535286	Completed	CLL, RS	27	Umbralisib, ublituximab, TG-1501	Phase I	
NCT03263637	Completed	AML, ALL, CLL, RS, NHL, MM	44	AZD4573	Phase I	
NCT03321643	Recruiting	DLBCL, NHL, RS	24	Atezolizumab, gemcitabine, oxaliplatin, rituximab	Phase I	
NCT05176691	Not yet recruiting	CLL, NHL, MCL, MZL, LPL, FL, DLBCL, RS	168	HMPL-760	Phase I	
NCT05107674	Recruiting	Solid tumors, RS	336	NX-1607	Phase I	
NCT04892277	Not yet recruiting	R/R NHL, R/R CLL, RS	25	CAR-T, cyclophosphamide, fludarabine	Phase I	
NCT03479268	Recruiting	CLL. DLBCL, FL, MCL, MZL, NHL, RS	30	Ibrutinib, pevonedistat	Phase I	
NCT01254578	Completed	CLL, RS, AML, APL, ALCL & other lymphomas	17	Lenalidomide	Phase I	
NCT04771572	Recruiting	NHL, RS, MM, AML, ALL	100	LP-118	Phase I	
NCT04806035	Recruiting	CLL, RS, FL, MZL, DLBCL, MCL	60	TG-1801, ublituximab	Phase I	
NCT03833180	Recruiting	CLL, MCL, FL, MZL, DLBCL, RS, BL, ALL		Zilovertamab vedotin	Phase I	

*ALCL*, anaplastic large cell lymphoma; *ALL*, acute lymphoblastic leukemia; *AML*, acute myeloid leukemia; *APL*, acute promyelocytic leukemia; *BL*, Burkitt lymphoma; *CLL*, chronic lymphocytic leukemia; *DLBCL*, diffuse large B-cell lymphoma; *FL*, follicular lymphoma; *LPL*, lymphoplasmacystic lymphoma; *MCL*, mantle cell lymphoma; *MM*, multiple myeloma; *MZL*, marginal zone lymphoma; *NHL*, non-Hodgkin lymphoma; *PLL*, prolymphocytic leukemia; *PMBCL*, primary mediastinal B-cell lymphoma; *R/R*, relapse/refractory; *RS*, Richter’s syndrome

#### Stem cell transplantation

An alternative approach to chemo-immunotherapy is either the autologous or allogenic-SCT, which however can only be adopted in fit RS patients, who generally represent a minority of patients (10–15%) [30]. Clinical data showed that RS patients who underwent allogenic-SCT as post remission therapy had longer survival compared to patients who received no additional therapy or SCT as salvage therapy, with some difference in terms of OS depending on the cohort analyzed (estimated 3-year OS of 36% for allogenic-SCT and 59% for autologous-SCT) [20, 23, 30]. These results have been recently confirmed in different studies, revealing a 5-year OS of 58% and confirming the long-term efficacy of SCT [22, 31•, 32, 33]. Moreover, a meta-analysis of the existing medical literature focused on the clinical efficacy of allogenic-SCT, performed in 2020, highlighted an encouraging ORR of 79%, including 33% of complete responses, and an OS rate of 49% [34].

### Novel therapeutic approaches

#### Antibody-based therapies

##### Naked antibodies

**Fig. 1:**
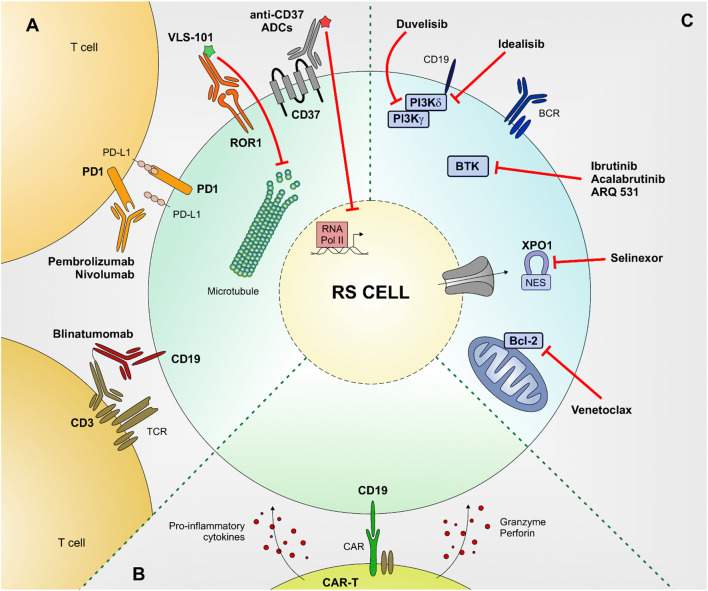
Schematic representation of novel therapeutic approaches to treat RS patients. The main novel therapeutic approaches to treat RS patients are summarized. Small molecules targeted therapies (**A**), antibody-based therapies (**B**), and CAR-T (**C**). Bcl-2 (B-cell lymphoma 2), XPO1 (exportin 1), BTK (Bruton tyrosine kinase), BCR (B-cell receptor), PI3K (phosphoinositide 3-kinase), ADC (antibody-drug conjugate), ROR1 (receptor tyrosine kinase-like orphan receptor 1), PD1 (programmed death 1), PD-L1 (programmed death 1 Ligand), TCR (T-cell receptor), RNA Pol II (RNA polymerase 2), CAR (chimeric antigen receptor), NES (nuclear export signal).

The programmed death 1 (PD-1) pathway plays a crucial role in tumor host immunity evasion and its blockade has emerged as an effective anti-cancer immunotherapy [35]. Preclinical studies suggested that PD-1 or PD-ligand 1 (PD-L1) blocking antibodies have efficacy in selected hematological malignancies, including DLBCL and other NHLs, all expressing high levels of these surface antigens [36, 37]. In addition, RS patients are characterized by a high expression of both PD-1 and PD-L1 [38, 39], opening for their targeting exploiting selective naked antibodies, capable of interfering with this signaling pathway (Fig. 1A).

Pembrolizumab is a PD-1 blocking antibody whose safety and clinical activity were initially evaluated in a phase II clinical trial that enrolled a small cohort of RS patients, most of them (approximately 70%) having been previously treated with ibrutinib, resulting in a 44% of ORR and 11 months of OS. This efficacy was associated to an acceptable safety profile, with only 20% of patients presenting severe hematologic toxicity [40]. These promising results were not overlapping the ones obtained by the KEYNOTE-170 multicenter phase II trial (NCT02576990) that enrolled relapsed/refractory RS patients. Indeed, in this subset of patients, pembrolizumab monotherapy resulted in a reduced clinical efficacy, with only 1 complete and 2 partial responses, leading to a 13% ORR [41].

Nivolumab is another PD-1-binding immune checkpoint inhibitor, capable of potentiating T-cell activity, that has already showed efficacy in several solid tumors [42–44]. The clinical impact of this humanized mAb, administered either alone or in combination with targeted agents (ibrutinib or venetoclax), has been evaluated in a small cohort of RS patients. Results were not encouraging with 90% of patients experiencing treatment failure, disease progression, and a median OS from the first dose of only 2 months [45].

Despite the low clinical efficacy observed with anti-PD-1 inhibitors in RS, different clinical trials are currently ongoing testing the impact of co-targeting strategies, where checkpoint inhibitors are administered together with molecules targeting critical signaling pathways for B cells, such as BTK or PI3K (NCT04271956, NCT02535286; Table 1).

##### Drug-conjugated antibodies

A promising therapeutic strategy for cancer treatment is based on antibody-drug conjugates (ADCs), engineered therapeutics combining the selectivity of a mAb, that recognizes a tumor-associated antigen, to the cytotoxicity of a payload. Their success depends on their effectiveness and the lack of off-target toxicities [46, 47]. Several ADCs are currently approved by FDA or in late-stage clinical development for treatment of both solid tumor and hematological malignancies [48•].

The clinical impact of ADCs in RS has been recently explored by our group, taking advantage of four RS patient–derived xenograft models [49], and targeting two molecules that are highly and selectively expressed by these neoplastic cells. Firstly, we explored the effects of VLS-101, an ADC comprising UC-961, a mAb targeting the extracellular domain of receptor tyrosine kinase-like orphan receptor 1 (ROR1) [50], linked to the anti-microtubule agent monomethyl auristatin E (MMAE). ROR1 is expressed by CLL cells and other cancers but not by healthy adult tissues, making it an attractive tumor-specific therapeutic target [51, 52]. Once VLS-101 is bound to its target, the complex is internalized and delivered to lysosomes, where MMAE is released via proteolytic cleavage and free to inhibit cell-cycle progression and to induce apoptosis of the target cell. VLS-101 has shown promising efficacy to treat RS, resulting in cell-cycle arrest and apoptosis ex vivo, and significantly reducing tumor burden in vivo resulting in a prolonged animal survival. Moreover, VLS-101 was characterized by a high selectivity since no clinical effects were registered in a ROR1-negative model and no adverse toxic effects showed in treated mice [53]. Based on these promising results, a phase I clinical trial testing VLS-101 in RS and other aggressive hematological malignancies is currently ongoing (NCT03833180).

The second target we explored in RS to be targeted with ADC is CD37, a surface molecule belonging to the tetraspanin family, showing a peculiar pattern of expression. Indeed, it is expressed by mature B and transformed leukemic/lymphoma cells, but not on normal T, NK, pro-B, and plasma cells. Given this expression profile, CD37 has been recently proposed as an actionable target for the treatment of CLL and non-Hodgkin lymphomas [54–58].

RS cells, both primary samples and PDX models, are characterized by CD37 surface expression, comparable to the one detected in DLBCL and follicular lymphoma cells. Three different anti-CD37 ADCs were tested, all generated using amanitin as a payload, a toxin that once internalized in the target cell is released from the mAb and free to localize to the nucleus where it binds to the RNA polymerase II, finally inhibiting messenger RNA synthesis. These compounds showed high selectivity and specificity as no targeting and toxic effects were highlighted in CD37-negative cells. Treatment of RS cells with these compounds, both ex vivo and in vivo, induced apoptosis and significantly prolonged survival of treated mice, after a single-dose administration of these ADCs, making CD37 an interesting target for RS patients (Fig. 1A) [59].

##### Bispecific antibodies

Bispecific monoclonal antibodies (bsAbs) are designed to bind two different epitopes or antigens, frequently expressed by distinct cells, and have been largely explored to drive an effector to a target cell [60•]. This approach has been recently adopted for the treatment of different B-cell malignancies [61•], including RS, where a refractory patient underwent a rapid and complete response following therapy with the bispecific anti-CD19/CD3 antibody blinatumomab, opening the way for a clinical trial (NCT03121534) with the aim of testing its efficacy and safety in RS (Fig. 1A) [62].

#### Chimeric antigen receptor T-cell therapy (CAR-T)

An innovative approach that has recently entered in the onco-hematological field is the targeting of CD19, an antigen exclusively expressed on normal and pathological B cells, via CAR-T [63, 64]. CAR-T cell therapy is designed to get T cells to fight against cancer prior ex vivo genetic manipulation of the receptor to better identify cancer antigens (Fig. 1B).

The first attempts of RS treatment with this approach were conducted few years ago in 2 patients with poor responses, including disease progression [65] and evolution to plasmablastic lymphoma [66]. Additional studies performed in relapsed patients who underwent hematopoietic stem cell transplantation and chemo-immunotherapy showed only partial responses [67, 68]. More recently, different trials based on larger cohorts of RS patients obtained quite satisfactory results, reaching an ORR of 71% and 56%, 4 weeks after cell therapy. Despite these results, a lower antitumor activity was registered in the subset of patients presenting large lymph node burden compared to those with a lower lymph node bulk [69–71]. In one of this study, CAR-T therapy was administered in combination with ibrutinib resulting in an increased clinical efficacy [71]. In line with these encouraging data, in a recent study that included 9 RS patients, heavily pretreated with chemo-immunotherapy, ibrutinib or ibrutinib in combination with venetoclax, an ORR of 90% with 5 patients showing a complete response in a median follow-up of 6 months was obtained [72].

Taken together, these results are encouraging, but prospective studies with larger cohorts of patients are needed to better understand how CAR-T can be combined with the available targeted therapies, which the patients who can benefit the most from this approach.

#### Small molecules–targeted therapy

Chemotherapy is one of the most effective therapeutic options against cancer, even though it is accompanied by several side effects. A breakthrough in this field has been the introduction of targeted therapies, which are based on pharmacological agents capable of selectively interfering with proteins involved in tumorigenesis. Focusing on molecular changes that are uniquely associated to a specific type of cancer may lead to improved therapeutic benefits, accompanied by a more safety profile, tailoring treatments to an individual patient’s tumor. Besides mAbs and ADCs, targeted therapy may involve the use of small molecule inhibitors, drugs capable of recognizing and block kinases, epigenetic regulatory proteins, and DNA damaged repair enzymes and proteasome [73].

Hematological malignancies have been an example of how small molecule inhibitors can be successfully adopted in the clinics [74] and treatment of CLL has been revolutionized by the introduction of BTK, PI3K, and Bcl-2 inhibitors [75].

These small molecule inhibitors have started to be translated in the clinic for the treatment of RS patients, even though results are still limited and sometimes discouraging (Fig. 1C).

Ibrutinib, a BTK inhibitor, has shown well tolerability in small cohorts of patients but poor clinical effects with only partial responses and a complete response obtained only in one patient [76–79]. Similar limited results have been obtained also with acalabrutinib, an irreversible BTK inhibitor, that showed a good tolerability profile, but poor responses when used as monotherapy [80]. Clinical data in RS patients are still missing for ARQ 531, another reversible non-specific BTK inhibitor, that has been tested in murine models of CLL and aggressive B-cell lymphomas [81], resulting in a prolonged survival of mice compared to animals treated with ibrutinib [82]. An ongoing phase I trial (NCT03162536) is enrolling patients, including RS patients, for testing safety, tolerability, and efficacy of ARQ 531 [83, 84].

AKT, another element of the BCR signaling cascade downstream to the PI3K kinase, has been shown to be active in RS and its constitutive activation in the Eμ-TCL1 CLL model can induce an aggressive lymphoma that mimics the clinical features of RS, suggesting that PI3K/AKT kinases play a key role in RS transformation [85, 86]. Idelalisib is a PI3Kδ inhibitor that has shown great clinical activity in CLL despite a significant toxicity, even in relapsed/refractory patients, when combined either with rituximab or with bendamustine regimens [75]. When administered to RS patients in a small cohort trial (4 patients), it has demonstrated some activity with no disease progression in all patients, one complete response and two partial responses [87]. Moreover, an independent case report confirmed idelalisib efficacy in RS, with a complete response achieved in 3 weeks, even though patient relapsed rapidly after drug discontinuation due to severe side effects [88].

More recently, our group has shown preclinical efficacy of duvelisib, a dual PI3Kδ/γ inhibitor, in PI3K expressing RS-PDX models [85]. Inhibition of the PI3K signaling pathway resulted in AKT downregulation and GSK3β activation, leading to ubiquitination and subsequent degradation of both c-Myc and Mcl-1, finally resulting in an increased apoptotic rate of RS cells. Moreover, treatment of RS-PDX mice resulted in a significant reduction of tumor volume and in a prolonged survival of mice, while no effects were obtained in RS cells with a reduced PI3Kγ expression underlining the high selectivity of this compound [85]. These encouraging results opened for further investigation on PI3K inhibition in RS patients and provided a rationale for a clinical trial (NCT03892044) investigating the clinical activity of duvelisib together with the anti-PD1 nivolumab. Moreover, given the selective activity in PI3K expressing cells, these data prompt in favor of a molecular profiling of RS patients prior to treatment decision to identify in advance those who will benefit the most from a specific targeted drug administration.

Another interesting molecular target is represented by the anti-apoptotic protein Bcl-2, whose targeting by Venetoclax, an orally bioavailable BH3 mimetic, in CLL patients have shown high percentage of durable responses, even in patients carrying the chromosome 17p deletion [18, 89]. When tested in RS-PDX models, venetoclax showed efficacy in Bcl-2-expressing cells, inducing apoptosis and prolonging mice survival [85]. However, clinical trials testing its efficacy in RS patients as monotherapy resulted only in partial responses [90, 91].

Lastly, one of the small molecule inhibitors that has increasingly gained attention in cancer treatment in the last years is selinexor, a selective inhibitor of nuclear export protein XPO1 [92]. Indeed, protein transport across nuclear membrane is often dysregulated in cancers [93] and *XPO1* has been shown to be overexpressed and/or mutated in several hematological malignancies, including CLL and RS, thus representing an interesting target [94, 95]. In a phase I pilot study, including 6 refractory/relapsed RS patients, selinexor used in monotherapy was generally well tolerated and induced partial response in 2 out 5 patients [96]. However, no additional studies are available thus its efficacy in RS remains to be determined and better explored (Fig. 1C).

#### Combination strategies

CLL therapy and clinical responses have radically changed since the introduction of small molecules replacing traditional chemo-immunotherapy approaches [75]. However, as discussed above, many of these novel compounds are associated with poor or partial responses in RS, likely due to a more aggressive behavior of these cells even because of a more complex karyotype or genetic background. Therefore, combination of drugs targeting different molecules or molecular pathways can be envisage as an effective strategy to overcome resistance.

Ibrutinib has been already tested in combination with several other agents. In 2015, Lamar and colleagues reported of a RS patient, heavily treated with chemo-immunotherapy before and after transformation, who experienced a significant, but unfortunately temporary, reduction of tumor burden in almost all infiltrated lymph nodes within 1 month of ibrutinib and rituximab treatment [97]. Similar results have been obtained in 3 patients treated with ibrutinib and ofatumumab, another anti-CD20 monoclonal antibody (NCT01217749). Two of them had a stable disease for a median time of 10 months, while the other had a partial response, before undergoing disease progression 5 months later [98]. Finally, BTK inhibition has been tested in combination with the anti-PD-1 agent nivolumab, in a trial that included patients with different relapsed/refractory B-cell hematological malignancies along with 20 RS cases. The best clinical responses were obtained in the RS cohort, with an ORR of 65% and two patients experiencing complete remission. Due to adverse events in a significant proportion of patients, treatment was discontinued, but the promising results support for further clinical assessment [99].

Similar combination trials have been proposed for acalabrutinib and other BTK inhibitors. In 2019, Appleby and colleagues has started the STELLAR trial protocol (NCT03899337), a prospective phase II randomized study of R-CHOP alone or in combination with acalabrutinib in a large cohort of RS patients. Results from this trial will highlight the safety, feasibility, and clinical activity of the addition of acalabrutinib to standard R-CHOP for RS [100]. Recently, the novel BTK inhibitor DTRM-12 has been tested in combination with the mTOR inhibitor everolimus and pomalidomide in RS, exploring the potential synthetic lethality of this therapeutic setting (NCT04305444). This combination had an acceptable safety profile and resulted in an ORR of 45%, and it is now investigated in a phase II expansion study [101].

In the last couple of years, preliminary results on combination strategies including the Bcl-2 inhibitor venetoclax are coming to the stage for RS treatment. In a phase II trial (NCT03054896), Davids and colleagues evaluated the therapeutic response of venetoclax in combination with chemo-immunotherapy regimen based on R-EPOCH. On a cohort of 26 patients, 13 achieved CR and 3 a partial response, with an ORR of 62% and a median OS of 19.6 months, with neutropenia and thrombocytopenia as major toxic effects [102•].

Encouraging data are also coming from preclinical model of RS. We have recently showed that the dual targeting of Bcl-2 and PI3K, through the combination of venetoclax and duvelisib, synergistically induced apoptosis in target expressing cells both ex vivo and in vivo in RS-PDX models, blocking tumor growth and significantly prolonging mice survival, even compared to each drug alone. The molecular mechanism beneath this effect relies on the concomitant inactivation of Mcl-1, c-Myc, and Bcl-2, via GSK3β activation [85]. Similar results were obtained combining venetoclax with other BH3 mimetics or with bromodomain extra-terminal (BET) protein targeting chimera (PROTAC), bifunctional molecules capable of hijacking the ubiquitin-proteasome system to induce degradation of BET proteins [103], suggesting that the simultaneous targeting of key molecular players within RS cells may represents a winning strategy [104].

Understanding the genetics and biology of RS are necessary steps in deciphering the critical pathways these cells rely on and identifying target(s). Despite advancements in the treatment options, based on different targeting approaches, RS remains a disease asking for effective therapies. Novel insights for therapeutic opportunities are expected to come in the next years when results from several clinical trials as well as from experimental models will be available and will finally reach patients’ bedside.

## References

[1] Swerdlow SHCE, Harris NL, Jaffe ES, Pileri SA, Stein H, Thiele J. WHO classification of tumours of haematopoietic and lymphoid tissues, vol 2 edn 4th; 2017.

[2] Chapuy B, Stewart C, Dunford AJ, Kim J, Kamburov A, Redd RA, Lawrence MS, Roemer MGM, Li AJ, Ziepert M (2018). Molecular subtypes of diffuse large B cell lymphoma are associated with distinct pathogenic mechanisms and outcomes. Nat Med.

[3] Reddy A, Zhang J, Davis NS, Moffitt AB, Love CL, Waldrop A, Leppa S, Pasanen A, Meriranta L, Karjalainen-Lindsberg ML (2017). Genetic and functional drivers of diffuse large B cell lymphoma. Cell.

[4] Schmitz R, Wright GW, Huang DW, Johnson CA, Phelan JD, Wang JQ, Roulland S, Kasbekar M, Young RM, Shaffer AL (2018). Genetics and pathogenesis of diffuse large B-cell lymphoma. N Engl J Med.

[5] Song JY, Yu J, Chan WC (2015). Gene expression profiling in non-Hodgkin lymphomas. Cancer Treat Res.

[6] Parikh SA, Rabe KG, Call TG, Zent CS, Habermann TM, Ding W, Leis JF, Schwager SM, Hanson CA, Macon WR (2013). Diffuse large B-cell lymphoma (Richter syndrome) in patients with chronic lymphocytic leukaemia (CLL): a cohort study of newly diagnosed patients. Br J Haematol.

[7] Rossi D, Cerri M, Capello D, Deambrogi C, Rossi FM, Zucchetto A, De Paoli L, Cresta S, Rasi S, Spina V (2008). Biological and clinical risk factors of chronic lymphocytic leukaemia transformation to Richter syndrome. Br J Haematol.

[8] Fayad L, Robertson LE, O'Brien S, Manning JT, Wright S, Hagemeister F, Cabanillas F, Keating MJ (1996). Hodgkin’s disease variant of Richter’s syndrome: experience at a single institution. Leuk Lymphoma.

[9] Jamroziak K, Grzybowska-Izydorczyk O, Jesionek-Kupnicka D, Gora-Tybor J, Robak T (2012). Poor prognosis of Hodgkin variant of Richter transformation in chronic lymphocytic leukemia treated with cladribine. Br J Haematol.

[10] Rossi D, Spina V, Deambrogi C, Rasi S, Laurenti L, Stamatopoulos K, Arcaini L, Lucioni M, Rocque GB, Xu-Monette ZY (2011). The genetics of Richter syndrome reveals disease heterogeneity and predicts survival after transformation. Blood.

[11] Hallek M, Al-Sawaf O (2021). Chronic lymphocytic leukemia: 2022 update on diagnostic and therapeutic procedures. Am J Hematol.

[12] Robak T, Blonski JZ, Gora-Tybor J, Kasznicki M, Konopka L, Ceglarek B, Komarnicki M, Lewandowski K, Hellmann A, Lewandowski K (2004). Second malignancies and Richter’s syndrome in patients with chronic lymphocytic leukaemia treated with cladribine. Eur J Cancer.

[13] Solh M, Rai KR, Peterson BL, Kolitz JE, Appelbaum FR, Tallman MS, Belch A, Larson RA, Morrison VA (2013). The impact of initial fludarabine therapy on transformation to Richter syndrome or prolymphocytic leukemia in patients with chronic lymphocytic leukemia: analysis of an intergroup trial (CALGB 9011). Leuk Lymphoma.

[14] Ahn IE, Underbayev C, Albitar A, Herman SE, Tian X, Maric I, Arthur DC, Wake L, Pittaluga S, Yuan CM (2017). Clonal evolution leading to ibrutinib resistance in chronic lymphocytic leukemia. Blood.

[15] Furman RR, Sharman JP, Coutre SE, Cheson BD, Pagel JM, Hillmen P, Barrientos JC, Zelenetz AD, Kipps TJ, Flinn I (2014). Idelalisib and rituximab in relapsed chronic lymphocytic leukemia. N Engl J Med.

[16] Munir T, Brown JR, O'Brien S, Barrientos JC, Barr PM, Reddy NM, Coutre S, Tam CS, Mulligan SP, Jaeger U (2019). Final analysis from RESONATE: up to six years of follow-up on ibrutinib in patients with previously treated chronic lymphocytic leukemia or small lymphocytic lymphoma. Am J Hematol.

[17] Roberts AW, Stilgenbauer S, Seymour JF, Huang DCS (2017). Venetoclax in patients with previously treated chronic lymphocytic leukemia. Clin Cancer Res.

[18] Stilgenbauer S, Eichhorst B, Schetelig J, Coutre S, Seymour JF, Munir T, Puvvada SD, Wendtner CM, Roberts AW, Jurczak W (2016). Venetoclax in relapsed or refractory chronic lymphocytic leukaemia with 17p deletion: a multicentre, open-label, phase 2 study. Lancet Oncol.

[19] Langerbeins P, Busch R, Anheier N, Durig J, Bergmann M, Goebeler ME, Hurtz HJ, Stauch MB, Stilgenbauer S, Dohner H (2014). Poor efficacy and tolerability of R-CHOP in relapsed/refractory chronic lymphocytic leukemia and Richter transformation. Am J Hematol.

[20] Kharfan-Dabaja MA, Kumar A, Stingo FE, Khimani F, Hussaini M, Ayala E, Nishihori T, Shah B, Locke FL, Pinilla-Ibarz J (2018). Allogeneic hematopoietic cell transplantation for Richter syndrome: a single-center experience. Clin Lymphoma Myeloma Leuk.

[21] Herrera AF, Ahn KW, Litovich C, Chen Y, Assal A, Bashir Q, Bayer RL, Coleman M, DeFilipp Z, Farhadfar N (2021). Autologous and allogeneic hematopoietic cell transplantation for diffuse large B-cell lymphoma-type Richter syndrome. Blood Adv.

[22] Abrisqueta P, Delgado J, Alcoceba M, Oliveira AC, Loscertales J, Hernandez-Rivas JA, Ferra C, Cordoba R, Yanez L, Medina A (2020). Clinical outcome and prognostic factors of patients with Richter syndrome: real-world study of the Spanish Chronic Lymphocytic Leukemia Study Group (GELLC). Br J Haematol.

[23] Tsimberidou AM, O'Brien S, Khouri I, Giles FJ, Kantarjian HM, Champlin R, Wen S, Do KA, Smith SC, Lerner S (2006). Clinical outcomes and prognostic factors in patients with Richter’s syndrome treated with chemotherapy or chemoimmunotherapy with or without stem-cell transplantation. J Clin Oncol.

[24] Tadmor T, Shvidel L, Bairey O, Goldschmidt N, Ruchlemer R, Fineman R, Rahimi-Levene N, Herishanu Y, Yuklea M, Arad A (2014). Richter’s transformation to diffuse large B-cell lymphoma: a retrospective study reporting clinical data, outcome, and the benefit of adding rituximab to chemotherapy, from the Israeli CLL Study Group. Am J Hematol.

[25] Eyre TA, Clifford R, Bloor A, Boyle L, Roberts C, Cabes M, Collins GP, Devereux S, Follows G, Fox CP (2016). NCRI phase II study of CHOP in combination with ofatumumab in induction and maintenance in newly diagnosed Richter syndrome. Br J Haematol.

[26] Wierda WG, Kipps TJ, Mayer J, Stilgenbauer S, Williams CD, Hellmann A, Robak T, Furman RR, Hillmen P, Trneny M (2010). Ofatumumab as single-agent CD20 immunotherapy in fludarabine-refractory chronic lymphocytic leukemia. J Clin Oncol.

[27] Rogers KA, Huang Y, Ruppert AS, Salem G, Stephens DM, Heerema NA, Andritsos LA, Awan FT, Byrd JC, Flynn JM (2018). A single-institution retrospective cohort study of first-line R-EPOCH chemoimmunotherapy for Richter syndrome demonstrating complex chronic lymphocytic leukaemia karyotype as an adverse prognostic factor. Br J Haematol.

[28] Tsimberidou AM, Kantarjian HM, Cortes J, Thomas DA, Faderl S, Garcia-Manero G, Verstovsek S, Ferrajoli A, Wierda W, Alvarado Y (2003). Fractionated cyclophosphamide, vincristine, liposomal daunorubicin, and dexamethasone plus rituximab and granulocyte-macrophage-colony stimulating factor (GM-CSF) alternating with methotrexate and cytarabine plus rituximab and GM-CSF in patients with Richter syndrome or fludarabine-refractory chronic lymphocytic leukemia. Cancer.

[29] Dabaja BS, O'Brien SM, Kantarjian HM, Cortes JE, Thomas DA, Albitar M, Schlette ES, Faderl S, Sarris A, Keating MJ (2001). Fractionated cyclophosphamide, vincristine, liposomal daunorubicin (daunoXome), and dexamethasone (hyperCVXD) regimen in Richter’s syndrome. Leuk Lymphoma.

[30] Cwynarski K, van Biezen A, de Wreede L, Stilgenbauer S, Bunjes D, Metzner B, Koza V, Mohty M, Remes K, Russell N (2012). Autologous and allogeneic stem-cell transplantation for transformed chronic lymphocytic leukemia (Richter’s syndrome): a retrospective analysis from the chronic lymphocytic leukemia subcommittee of the chronic leukemia working party and lymphoma working party of the European Group for Blood and Marrow Transplantation. J Clin Oncol.

[31] Al-Sawaf O, Robrecht S, Bahlo J, Fink AM, Cramer P, Tresckow JV, Lange E, Kiehl M, Dreyling M, Ritgen M (2021). Richter transformation in chronic lymphocytic leukemia (CLL)-a pooled analysis of German CLL Study Group (GCLLSG) front line treatment trials. Leukemia.

[32] Kim HT, Baker PO, Parry E, Davids M, Alyea EP, Ho VT, Cutler C, Koreth J, Gooptu M, Romee R (2021). Allogeneic hematopoietic cell transplantation outcomes in patients with Richter’s transformation. Haematologica.

[33] Lahoud OB, Devlin SM, Maloy MA, Roeker LE, Dahi PB, Ponce DM, Gyurkocza B, Koehne G, Young JW, Castro-Malaspina HR (2021). Reduced-intensity conditioning hematopoietic stem cell transplantation for chronic lymphocytic leukemia and Richter’s transformation. Blood Adv.

[34] Aulakh S, Reljic T, Yassine F, Ayala E, Chavez JC, Chanan-Khan A, Pinilla-Ibarz J, Kumar A, Kharfan-Dabaja MA (2021). Allogeneic hematopoietic cell transplantation is an effective treatment for patients with Richter syndrome: a systematic review and meta-analysis. Hematol Oncol Stem Cell Ther.

[35] Dermani FK, Samadi P, Rahmani G, Kohlan AK, Najafi R (2019). PD-1/PD-L1 immune checkpoint: potential target for cancer therapy. J Cell Physiol.

[36] Lesokhin AM, Ansell SM, Armand P, Scott EC, Halwani A, Gutierrez M, Millenson MM, Cohen AD, Schuster SJ, Lebovic D (2016). Nivolumab in patients with relapsed or refractory hematologic malignancy: preliminary results of a phase Ib study. J Clin Oncol.

[37] Xu-Monette ZY, Zhou J, Young KH (2018). PD-1 expression and clinical PD-1 blockade in B-cell lymphomas. Blood.

[38] Behdad A, Griffin B, Chen YH, Ma S, Kelemen K, Lu X, Chen QC (2019). PD-1 is highly expressed by neoplastic B-cells in Richter transformation. Br J Haematol.

[39] He R, Ding W, Viswanatha DS, Chen D, Shi M, Van Dyke D, Tian S, Dao LN, Parikh SA, Shanafelt TD (2018). PD-1 expression in chronic lymphocytic leukemia/small lymphocytic lymphoma (CLL/SLL) and large B-cell Richter transformation (DLBCL-RT): a characteristic feature of DLBCL-RT and potential surrogate marker for clonal relatedness. Am J Surg Pathol.

[40] Ding W, LaPlant BR, Call TG, Parikh SA, Leis JF, He R, Shanafelt TD, Sinha S, Le-Rademacher J, Feldman AL (2017). Pembrolizumab in patients with CLL and Richter transformation or with relapsed CLL. Blood.

[41] Armand P, Murawski N, Molin D, Zain J, Eichhorst B, Gulbas Z, Hawkes EA, Pagel JM, Phillips T, Ribrag V (2020). Pembrolizumab in relapsed or refractory Richter syndrome. Br J Haematol.

[42] Koppolu V, Rekha Vasigala VK (2018). Checkpoint immunotherapy by nivolumab for treatment of metastatic melanoma. J Cancer Res Ther.

[43] Smith KM, Desai J (2018). Nivolumab for the treatment of colorectal cancer. Expert Rev Anticancer Ther.

[44] Rajan A, Kim C, Heery CR, Guha U, Gulley JL (2016). Nivolumab, anti-programmed death-1 (PD-1) monoclonal antibody immunotherapy: role in advanced cancers. Hum Vaccin Immunother.

[45] Rogers KA, Huang Y, Dotson E, Lundberg J, Andritsos LA, Awan FT, Woyach JA, Byrd JC (2019). Use of PD-1 (PDCD1) inhibitors for the treatment of Richter syndrome: experience at a single academic centre. Br J Haematol.

[46] Ceci C, Lacal PM, Graziani G (2022). Antibody-drug conjugates: Resurgent anticancer agents with multi-targeted therapeutic potential. Pharmacol Ther.

[47] Jabbour E, Paul S, Kantarjian H (2021). The clinical development of antibody-drug conjugates - lessons from leukaemia. Nat Rev Clin Oncol.

[48] Drago JZ, Modi S, Chandarlapaty S (2021). Unlocking the potential of antibody-drug conjugates for cancer therapy. Nat Rev Clin Oncol.

[49] Vaisitti T, Braggio E, Allan JN, Arruga F, Serra S, Zamo A, Tam W, Chadburn A, Furman RR, Deaglio S (2018). Novel Richter syndrome xenograft models to study genetic architecture, biology, and therapy responses. Cancer Res.

[50] Choi MY, Widhopf GF, Wu CC, Cui B, Lao F, Sadarangani A, Cavagnaro J, Prussak C, Carson DA, Jamieson C (2015). Pre-clinical specificity and safety of UC-961, a first-in-class monoclonal antibody targeting ROR1. Clin Lymphoma Myeloma Leuk.

[51] Daneshmanesh AH, Porwit A, Hojjat-Farsangi M, Jeddi-Tehrani M, Tamm KP, Grander D, Lehmann S, Norin S, Shokri F, Rabbani H (2013). Orphan receptor tyrosine kinases ROR1 and ROR2 in hematological malignancies. Leuk Lymphoma.

[52] Zhang S, Chen L, Wang-Rodriguez J, Zhang L, Cui B, Frankel W, Wu R, Kipps TJ (2012). The onco-embryonic antigen ROR1 is expressed by a variety of human cancers. Am J Pathol.

[53] Vaisitti T, Arruga F, Vitale N, Lee TT, Ko M, Chadburn A, Braggio E, Di Napoli A, Iannello A, Allan JN (2021). ROR1 targeting with the antibody-drug conjugate VLS-101 is effective in Richter syndrome patient-derived xenograft mouse models. Blood.

[54] Deckert J, Park PU, Chicklas S, Yi Y, Li M, Lai KC, Mayo MF, Carrigan CN, Erickson HK, Pinkas J (2013). A novel anti-CD37 antibody-drug conjugate with multiple anti-tumor mechanisms for the treatment of B-cell malignancies. Blood.

[55] Maaland AF, Heyerdahl H, O'Shea A, Eiriksdottir B, Pascal V, Andersen JT, Kolstad A, Dahle J (2019). Targeting B-cell malignancies with the beta-emitting anti-CD37 radioimmunoconjugate (177)Lu-NNV003. Eur J Nucl Med Mol Imaging.

[56] Pagel JM, Spurgeon SE, Byrd JC, Awan FT, Flinn IW, Lanasa MC, Eisenfeld AJ, Stromatt SC, Gopal AK (2015). Otlertuzumab (TRU-016), an anti-CD37 monospecific ADAPTIR() therapeutic protein, for relapsed or refractory NHL patients. Br J Haematol.

[57] Pereira DS, Guevara CI, Jin L, Mbong N, Verlinsky A, Hsu SJ, Avina H, Karki S, Abad JD, Yang P (2015). AGS67E, an anti-CD37 monomethyl auristatin E antibody-drug conjugate as a potential therapeutic for B/T-cell malignancies and AML: a new role for CD37 in AML. Mol Cancer Ther.

[58] Repetto-Llamazares AH, Larsen RH, Patzke S, Fleten KG, Didierlaurent D, Pichard A, Pouget JP, Dahle J (2015). Targeted cancer therapy with a novel anti-CD37 beta-particle emitting radioimmunoconjugate for treatment of non-Hodgkin lymphoma. PLoS One.

[59] Vaisitti T, Vitale N, Iannello A, Brandimarte L, Micillo M, Papotti MG, Di Napoli A, Orlik C, Kulke M, Pahl A, Deaglio S (2021). Anti-CD37 alpha-aminitin conjugated antibodies as therapeutic weapons for Richter’s syndrome. Blood.

[60] Labrijn AF, Janmaat ML, Reichert JM, Parren P (2019). Bispecific antibodies: a mechanistic review of the pipeline. Nat Rev Drug Discov.

[61] Lejeune M, Kose MC, Duray E, Einsele H, Beguin Y, Caers J (2020). Bispecific, T-cell-recruiting antibodies in B-cell malignancies. Front Immunol.

[62] Alderuccio JP, Mackrides N, Chapman JR, Vega F, Lossos IS (2019). Rapid complete response to blinatumomab as a successful bridge to allogeneic stem cell transplantation in a case of refractory Richter syndrome. Leuk Lymphoma.

[63] Holstein SA, Lunning MA (2020). CAR T-cell therapy in hematologic malignancies: a voyage in progress. Clin Pharmacol Ther.

[64] Lemal R, Tournilhac O (2019). State-of-the-art for CAR T-cell therapy for chronic lymphocytic leukemia in 2019. J Immunother Cancer.

[65] Xia L, Wang Y, Li T, Hu X, Chen Q, Liu L, Jiang B, Li C, Wang H, Wang S (2019). The clinical study on treatment of CD19-directed chimeric antigen receptor-modified T cells in a case of refractory Richter syndrome. Cancer Med.

[66] Evans A, Burack R, Rothberg P, Porter D, Liesveld J. Evolution to plasmablastic lymphoma (PBL) after CAR-T cell therapy in a case of SLL/CLL with Richter’s transformation. Blood 2014, ASH abstract 642.

[67] Cruz CR, Micklethwaite KP, Savoldo B, Ramos CA, Lam S, Ku S, Diouf O, Liu E, Barrett AJ, Ito S (2013). Infusion of donor-derived CD19-redirected virus-specific T cells for B-cell malignancies relapsed after allogeneic stem cell transplant: a phase 1 study. Blood.

[68] Kochenderfer JN, Dudley ME, Kassim SH, Somerville RP, Carpenter RO, Stetler-Stevenson M, Yang JC, Phan GQ, Hughes MS, Sherry RM (2015). Chemotherapy-refractory diffuse large B-cell lymphoma and indolent B-cell malignancies can be effectively treated with autologous T cells expressing an anti-CD19 chimeric antigen receptor. J Clin Oncol.

[69] Benjamini O, Shimoni A, Besser M, Shem-Tov N, Danylesko I, Yerushalmi R, Merkel D, Tadmor T, Lavie D, Fineman R, et al. Safety and efficacy of CD19-CAR T cells in Richter’s transformation after targeted therapy for chronic lymphocytic leukemia. Blood 2020, ASH abstract 545.

[70] Turtle CJ, Hay KA, Hanafi LA, Li D, Cherian S, Chen X, Wood B, Lozanski A, Byrd JC, Heimfeld S (2017). Durable molecular remissions in chronic lymphocytic leukemia treated with CD19-Specific chimeric antigen receptor-modified T cells after failure of ibrutinib. J Clin Oncol.

[71] Gauthier J, Hirayama AV, Purushe J, Hay KA, Lymp J, Li DH, Yeung CCS, Sheih A, Pender BS, Hawkins RM (2020). Feasibility and efficacy of CD19-targeted CAR T cells with concurrent ibrutinib for CLL after ibrutinib failure. Blood.

[72] Kittai AS, Bond DA, William B, Saad A, Penza S, Efebera Y, Larkin K, Wall SA, Choe HK, Bhatnagar B (2020). Clinical activity of axicabtagene ciloleucel in adult patients with Richter syndrome. Blood Adv.

[73] Zhong L, Li Y, Xiong L, Wang W, Wu M, Yuan T, Yang W, Tian C, Miao Z, Wang T (2021). Small molecules in targeted cancer therapy: advances, challenges, and future perspectives. Signal Transduct Target Ther.

[74] Sochacka-Cwikla A, Maczynski M, Regiec A. FDA-approved drugs for hematological malignancies-the last decade review. Cancers (Basel). 2021;14. 10.3390/cancers14010087 PMC8750348 35008250

[75] Scheffold A, Stilgenbauer S (2020). Revolution of chronic lymphocytic leukemia therapy: the chemo-free treatment paradigm. Curr Oncol Rep.

[76] Giri S, Hahn A, Yaghmour G, Martin MG (2015). Ibrutinib has some activity in Richter’s syndrome. Blood Cancer J.

[77] Master S, Leary C, Takalkar A, Coltelingam J, Mansour R, Mills GM, Koshy N (2017). Successful treatment of Richter transformation with ibrutinib in a patient with chronic lymphocytic leukemia following allogeneic hematopoietic stem cell transplant. Case Rep Oncol.

[78] Tsang M, Shanafelt TD, Call TG, Ding W, Chanan-Khan A, Leis JF, Nowakowski GS, Bowen D, Conte M, Schwager SM (2015). The efficacy of ibrutinib in the treatment of Richter syndrome. Blood.

[79] Fischer A, Bastian S, Cogliatti S, Mey U, Saub J, Schanz U, Padberg B, Hohloch K (2018). Ibrutinib-induced rapid response in chemotherapy-refractory Richter’s syndrome. Hematol Oncol.

[80] Eyre TA, Schuh A, Wierda WG, Brown JR, Ghia P, Pagel JM, Furman RR, Cheung J, Hamdy A, Izumi R (2021). Acalabrutinib monotherapy for treatment of chronic lymphocytic leukaemia (ACE-CL-001): analysis of the Richter transformation cohort of an open-label, single-arm, phase 1-2 study. Lancet Haematol.

[81] Rogers KA, El-Gamal D, Bonnie HK, Zachary HA, Virginia GM, Rose M, Smith LL, Yu L, Johnson AJ, Byrd JC, Lapalombella R, Woyach JA. The Eμ-Myc/TCL1 transgenic mouse as a new aggressive B-cell malignancy model suitable for preclinical therapeutics testing. Blood 2015;126.

[82] Reiff SD, Mantel R, Smith LL, Greene JT, Muhowski EM, Fabian CA, Goettl VM, Tran M, Harrington BK, Rogers KA (2018). The BTK inhibitor ARQ 531 targets ibrutinib-resistant CLL and Richter transformation. Cancer Discov.

[83] Woyach JA, Stephens DM, Flinn IW, Bhat SA, Savage R, Chai F, Eathiraj S, Granlund L, Szuszkiewicz L, Byrd JC (2019). A phase I dose escalation study of ARQ 531 in patients with relapsed or refractory B-cell lymphoid malignancies. EHA library.

[84] Woyach J, Stephens DM, Flinn IW, Bhat SA, Savage RE, Chai F, Eathiraj S, Granlund L, Szuszkiewicz LA, Schwartz B, Byrd JC. Final results of phase 1, dose escalation study evaluating ARQ 531 in patients with relapsed or refractory B-cell lymphoid malignancies. Blood 2019;134.

[85] Iannello A, Vitale N, Coma S, Arruga F, Chadburn A, Di Napoli A, Laudanna C, Allan JN, Furman RR, Pachter JA (2021). Synergistic efficacy of the dual PI3K-delta/gamma inhibitor duvelisib with the Bcl-2 inhibitor venetoclax in Richter syndrome PDX models. Blood.

[86] Kohlhaas V, Blakemore SJ, Al-Maarri M, Nickel N, Pal M, Roth A, Hovelmeyer N, Schafer SC, Knittel G, Lohneis P (2021). Active Akt signaling triggers CLL toward Richter transformation via overactivation of Notch1. Blood.

[87] Visentin A, Imbergamo S, Scomazzon E, Pravato S, Frezzato F, Bonaldi L, Pizzi M, Vio S, Gregianin M, Burei M (2019). BCR kinase inhibitors, idelalisib and ibrutinib, are active and effective in Richter syndrome. Br J Haematol.

[88] Bagacean C, Zdrenghea M, Saad H, Berthou C, Renaudineau Y, Tempescul A (2019). Rapid and complete response to idelalisib in a case of Richter syndrome. Onco Targets Ther.

[89] Stilgenbauer S, Eichhorst B, Schetelig J, Hillmen P, Seymour JF, Coutre S, Jurczak W, Mulligan SP, Schuh A, Assouline S (2018). Venetoclax for patients with chronic lymphocytic leukemia with 17p deletion: results from the full population of a phase II pivotal trial. J Clin Oncol.

[90] Davids MS, Roberts AW, Seymour JF, Pagel JM, Kahl BS, Wierda WG, Puvvada S, Kipps TJ, Anderson MA, Salem AH (2017). Phase I first-in-human study of venetoclax in patients with relapsed or refractory non-Hodgkin lymphoma. J Clin Oncol.

[91] Bouclet F, Calleja A, Dilhuydy MS, Veronese L, Pereira B, Amorim S, Cymbalista F, Herbaux C, de Guibert S, Roos-Weil D (2021). Real-world outcomes following venetoclax therapy in patients with chronic lymphocytic leukemia or Richter syndrome: a FILO study of the French compassionate use cohort. Ann Hematol.

[92] Walker JS, Garzon R, Lapalombella R (2020). Selinexor for advanced hematologic malignancies. Leuk Lymphoma.

[93] Taylor J, Sendino M, Gorelick AN, Pastore A, Chang MT, Penson AV, Gavrila EI, Stewart C, Melnik EM, Herrejon Chavez F (2019). Altered nuclear export signal recognition as a driver of oncogenesis. Cancer Discov.

[94] Stamatopoulos B, Antoniou P, Vavoulis D, Eyre TA, Clifford R, Cabes M, Dreau H, Schuh A. Characterization of recurrent mutations in patient with a Richter syndrome by targeted next generation sequencing. Blood 2016;128.

[95] Walker JS, Hing ZA, Harrington B, Baumhardt J, Ozer HG, Lehman A, Giacopelli B, Beaver L, Williams K, Skinner JN (2021). Recurrent XPO1 mutations alter pathogenesis of chronic lymphocytic leukemia. J Hematol Oncol.

[96] Kuruvilla J, Byrd JC, Flynn JM, Garzon R, Porcu P, Norori S, Savona M, Rashal T, Mirza M, Kauffman M (2014). The oral selective inhibitor of nuclear export (SINE) selinexor (KPT-330) demonstrates broad and durable clinical activity in relapsed / refractory non Hodgkin’s lymphoma (NHL). Blood.

[97] Lamar Z, Kennedy L, Kennedy B, Lynch M, Goad A, Hurd D, McIver Z (2015). Ibrutinib and rituximab induced rapid response in refractory Richter syndrome. Clin Case Rep.

[98] Jaglowski SM, Jones JA, Nagar V, Flynn JM, Andritsos LA, Maddocks KJ, Woyach JA, Blum KA, Grever MR, Smucker K (2015). Safety and activity of BTK inhibitor ibrutinib combined with ofatumumab in chronic lymphocytic leukemia: a phase 1b/2 study. Blood.

[99] Younes A, Brody J, Carpio C, Lopez-Guillermo A, Ben-Yehuda D, Ferhanoglu B, Nagler A, Ozcan M, Avivi I, Bosch F (2019). Safety and activity of ibrutinib in combination with nivolumab in patients with relapsed non-Hodgkin lymphoma or chronic lymphocytic leukaemia: a phase 1/2a study. Lancet Haematol.

[100] Appleby N, Eyre TA, Cabes M, Jackson A, Boucher R, Yates F, Fox S, Rawstron A, Hillmen P, Schuh A (2019). The STELLAR trial protocol: a prospective multicentre trial for Richter’s syndrome consisting of a randomised trial investigation CHOP-R with or without acalabrutinib for newly diagnosed RS and a single-arm platform study for evaluation of novel agents in relapsed disease. BMC Cancer.

[101] Mato AR, Schuster SJ, Foss FM, Isufi I, Ding W, Brander DM, Sitlinger A, Tun HW, Moustafa MA, Kennard K, King CM, Koehler A, Aitken C, He W, Kearney A, Gui M, Anderson BD, Rosenthal AC, Roeker LE, Huntington SF. A phase Ia/Ib study exploring the synthetic lethality of the orally administered novel BTK inhibitor, Dtrmwxhs-12 (DTRM-12), in combination with everolimus and pomalidomide in patients with relapsed/refractory CLL, DLBCL or other B-cell lymphomas. Blood 2019;134.

[102] Davids MS, Rogers KA, Tyekucheva S, Wang Z, Pazienza S, Renner SK, Montegaard J, Ihuoma U, Lehmberg TZ, Parry EM, Wu CJ, Jacobson CA, Fisher DC, Thompson PA, Brown JR (2022). Venetoclax plus dose-adjusted R-EPOCH for Richter syndrome. Blood.

[103] Zhou B, Hu J, Xu F, Chen Z, Bai L, Fernandez-Salas E, Lin M, Liu L, Yang CY, Zhao Y (2018). Discovery of a small-molecule degrader of bromodomain and extra-terminal (BET) proteins with picomolar cellular potencies and capable of achieving tumor regression. J Med Chem.

[104] Fiskus W, Mill CP, Perera D, Birdwell C, Deng Q, Yang H, Lara BH, Jain N, Burger J, Ferrajoli A (2021). BET proteolysis targeted chimera-based therapy of novel models of Richter transformation-diffuse large B-cell lymphoma. Leukemia.

[105] Tsimberidou AM, Wierda WG, Plunkett W, Kurzrock R, O'Brien S, Wen S, Ferrajoli A, Ravandi-Kashani F, Garcia-Manero G, Estrov Z (2008). Phase I-II study of oxaliplatin, fludarabine, cytarabine, and rituximab combination therapy in patients with Richter’s syndrome or fludarabine-refractory chronic lymphocytic leukemia. J Clin Oncol.

[106] Crombie JL, Tyekucheva S, Wang Z, Savell A, Brennan L, Lowney J, Francoeur K, Montegaard J, Kim AI, Soumerai JD, Arnason JE, Cruz AL, Berg S, Fisher DC, Brown JR, Davids MS (2020). Updated results from a phase I/II study of duvelisib and venetoclax in patients with relapsed or refractory CLL/SLL or Richter’s syndrome. Blood.

[107] Tsimberidou AM, Wierda WG, Wen S, Plunkett W, O'Brien S, Kipps TJ, Jones JA, Badoux X, Kantarjian H, Keating MJ (2013). Phase I-II clinical trial of oxaliplatin, fludarabine, cytarabine, and rituximab therapy in aggressive relapsed/refractory chronic lymphocytic leukemia or Richter syndrome. Clin Lymphoma Myeloma Leuk.

